# Research trends of mitochondrial dysfunction in hepatic fibrosis: a bibliometric analysis

**DOI:** 10.3389/fphys.2026.1767822

**Published:** 2026-02-27

**Authors:** Xingtao Jin, Yanlu Ma, Yiqiang Tang, Fengjie Qiao, Tong Xiao, Yu Cui, Man Li, Yueqiu Gao

**Affiliations:** 1 Institute of Cellular Immunity Laboratory, Shuguang Hospital, Shanghai University of Traditional Chinese Medicine, Shanghai, China; 2 Institute of Shanghai Key Laboratory of Traditional Chinese Clinical Medicine, Shanghai, China

**Keywords:** biomarker, hepatic fibrosis, mitochondria, mitochondrial dysfunction, non-alcoholic steatohepatitis

## Abstract

**Background:**

Hepatic fibrosis is a progressive pathological process driven by multiple chronic liver injury factors. Increasing evidence highlights that mitochondrial dysfunction serves as a pivotal mechanism in the onset and progression of hepatic fibrosis.

**Methods:**

A systematic search of the Web of Science Core Collection (WoSCC) and Scopus databases identified 1,634 relevant articles published between January 2005 and December 2025. Following the PRISMA guidelines, duplicate removal and quality control were performed. Bibliometric analysis tools including VOSviewer, CiteSpace, and Scimago Graphica were utilized to extract or calculate evaluation metrics, enabling analysis and visualization of knowledge maps. Publications were categorized by country, institution, author, journal, highly cited papers, and keywords. These variables were compared in terms of publication output and academic impact, including metrics such as citation counts, citation impact, H-index, and journal impact factor.

**Results:**

A total of 1,634 relevant publications were retrieved, originating from 92 countries or regions and 2858 research institutions. China and the United States led in both publication volume and impact; the most prolific institution was the Centro de Investigación Biomédica en Red (CIBER), followed by the University of California System. The International Journal of Molecular Sciences was the most frequently publishing journal, while Hepatology was the most highly cited journal. Heidari, Reza was the most prolific author; the five most cutting-edge keywords identified were oxidative stress, apoptosis, mitophagy, hepatic stellate cells, and reactive oxygen species. We confirmed three major research hotspots: activation of hepatic stellate cells, imbalance in mitochondrial quality control, and the vicious cycle of oxidative stress.

**Conclusion:**

Based on our previous discussions, mitochondria are increasingly recognized as central to the onset and progression of hepatic fibrosis. Related research is advancing rapidly and has become a key area for interdisciplinary collaboration. Future efforts should focus on: validating mitochondrial function biomarkers such as circulating mtDNA and mitochondria-specific metabolites; refining patient stratification based on mitochondrial dysfunction phenotypes (such as metabolic imbalance type, oxidative stress type); and advancing therapeutic strategies targeting mitochondrial quality control, metabolism, and redox balance. This will translate deep mechanistic insights into effective solutions for improving the clinical management of hepatic fibrosis.

## Introduction

Hepatic fibrosis represents an excessive wound-healing response following chronic liver injury, characterized by pathological accumulation of extracellular matrix. It may ultimately progress to cirrhosis, liver failure, and even hepatocellular carcinoma, emerging as a leading cause of morbidity and mortality worldwide (Wu et al., 2019; Morrison et al., 2022; Hong et al., 2016). Its etiology is diverse, encompassing viral hepatitis, alcoholic liver disease, non-alcoholic steatohepatitis (NASH), and autoimmune liver diseases. Despite differing etiologies, sustained activation of hepatic stellate cells represents a central hub in fibrosis development (Kim et al., 2019; Wan et al., 2017). Current therapeutic strategies primarily focus on controlling causative factors and symptomatic management, such as antiviral therapy and alcohol abstinence. However, specific targeted drugs capable of reversing fibrosis remain extremely limited, underscoring the urgent need to delve into its core molecular mechanisms for novel therapeutic development (Qu et al., 2018).

In recent years, research focus has shifted from solely examining extracellular matrix metabolism to delving into the intracellular signaling networks driving fibrosis. Mitochondria, traditionally regarded as the cell’s “powerhouse,” have been redefined as a pivotal signaling hub and regulatory node in the progression of hepatic fibrosis (Zhang et al., 2014). Under the stressful conditions of chronic liver injury, mitochondrial function undergoes profound disruption across multiple cell types—including hepatocytes, hepatic stellate cells, and macrophages—manifesting as uncoupled oxidative phosphorylation, excessive reactive oxygen species (ROS) production, impaired fatty acid β-oxidation, and dysregulated mitophagy (Daher-Abdi et al., 2021; Xu et al., 2022). This “mitochondrial dysfunction” is not merely a consequence of an energy crisis but an active driver propelling disease progression. For instance, excess ROS and damage-associated molecular patterns (DAMPs) originating from hepatocyte mitochondria can directly activate hepatic stellate cells, inducing their transformation into a pro-fibrotic myofibroblastic phenotype (Klepfish et al., 2020). Simultaneously, hepatic stellate cells undergo mitochondrial metabolic reprogramming—such as shifting toward aerobic glycolysis—which provides essential bioenergy and biosynthetic precursors for their proliferation, survival, and collagen synthesis (Yu et al., 2022). Furthermore, the accumulation of “damaged mitochondria” due to impaired mitochondrial clearance amplifies cellular injury and inflammatory signaling, creating a vicious cycle (Zhang et al., 2022).

Therefore, interventions targeting mitochondrial quality control, metabolic pathways, and redox balance are considered highly promising novel anti-fibrotic strategies. With the explosive growth of related research, this field has accumulated a vast body of mechanistic evidence and exhibits a high degree of interdisciplinarity. However, there remains a lack of systematic review of this rapidly evolving field to clearly map its knowledge structure, evolutionary trajectory, international collaboration models, and future frontiers.

Bibliometric analysis is a quantitative method for studying and evaluating academic literature. It effectively identifies emerging trends and evolutionary patterns within specific fields, providing valuable insights and evidence for assessing future research directions (He et al., 2024). This study conducts a visual analysis of research literature over the past 2 decades concerning the association between mitochondria and the mechanisms of hepatic fibrosis. It aims to explore the developmental trajectory, research hotspots, and emerging trends in this field, while projecting potential future focal directions. This analysis provides academic reference for deepening research into the role of mitochondria in hepatic fibrosis mechanisms and advancing clinical translation.

## Methods

### Data collection

WoSCC is renowned for its rigorous journal selection and reliable citation tracking, effectively capturing the impact and dissemination of scholarly work. Scopus excels in supporting interdisciplinary research through its extensive subject coverage and advanced citation tools. Combining WoSCC and Scopus provides a more comprehensive and accurate bibliometric analysis, offering deeper insights into research trends and academic developments. PubMed and EMBASE are established biomedical literature databases and play important roles in systematic reviews and clinical queries. However, for bibliometric analyses that rely heavily on citation tracking, collaboration mapping, and journal impact metrics, WoSCC and Scopus are more commonly adopted. To ensure that our dataset was representative of the core literature in this field, we performed a *post hoc* overlap check. A sample of highly cited articles and key journals identified from our WoSCC/Scopus dataset was cross-referenced with PubMed. This validation confirmed that the majority of influential publications were indexed across these databases, suggesting that our search strategy captured the central body of knowledge without omitting critical studies. Thus, while we did not systematically retrieve records from PubMed or EMBASE, the use of WoSCC and Scopus remains methodologically sound for the purposes of this bibliometric study.

**FIGURE 1:**
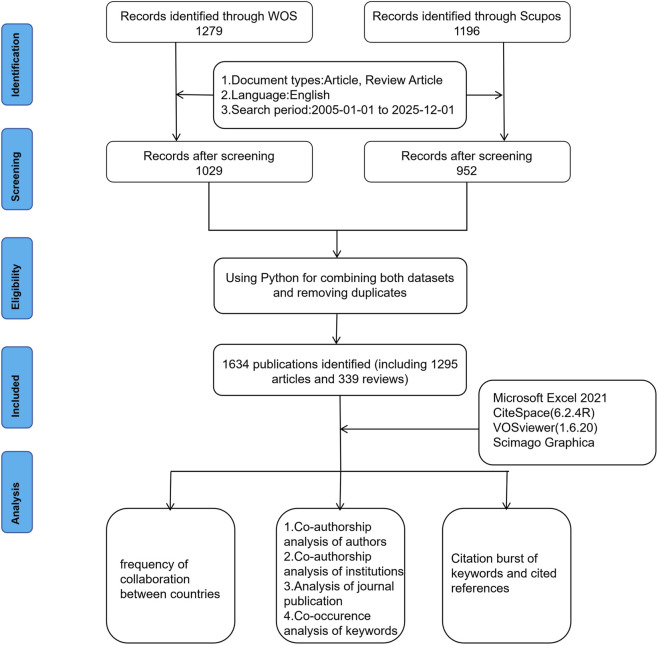
Flow-chart of the study.

Figure 1 provides detailed specifications of data retrieval and exclusion criteria. Initially, to obtain bibliometric data, we selected the WoSCC and Scopus databases as our primary research databases. For each database, we specifically designed search strategies, as detailed in Supplementary Tables S1-S2, which contain the exact search strings used. By combining free words and subject terms to enhance the sensitivity and specificity of the search. All subject terms originated from the Medical Subject Headings (MeSH) database. During the initial search, no language restrictions were set to minimize potential bias that might arise from excluding non-English publications. We retrieved relevant papers published between 1 January 2005 and 1 December 2025. To facilitate further content analysis, only articles and review articles were included, and the language was limited to English. Complete records and cited references were then extracted from relevant publications and saved in plain text format for subsequent analysis. Ultimately, WoSCC yielded 1,029 documents, while Scopus yielded 952 documents.

Following data retrieval from WoSCC and Scopus, a custom Python script was employed to merge and deduplicate the datasets, ensuring each publication was counted only once. The deduplication process first matched records based on identical Digital Object Identifiers (DOIs). For entries lacking a DOI, matches were established by comparing the normalized publication title—converted to lowercase with punctuation and extra whitespace removed—alongside the first author’s surname. All matches flagged by the script subsequently underwent manual review to confirm accuracy and resolve any discrepancies arising from formatting variations. This procedure yielded a final corpus of 1,634 unique publications. The full analysis pipeline, including the code for deduplication and subsequent bibliometric network construction, is openly accessible in a public repository (https://github.com/LeoMengTCM/scopus-wos-tools) to ensure transparency and reproducibility. After this quality control step, a total of 1,634 unique studies were retained for further analysis, comprising 1,295 articles and 339 reviews (1,029 from WoSCC, 605 from Scopus).

### Study selection

Two reviewers independently screened titles and abstracts, then assessed full texts against eligibility criteria. Discrepancies were resolved through discussion. After removing duplicates, 1,634 unique studies were included for further analysis.

### Bibliometric and scientometric analysis

The bibliometric tools employed in this study include VOSviewer (version 1.6.20), CiteSpace (version 6.2.4R), Scimago Graphica, and Microsoft Office Excel 2021. VOSviewer is a program designed to construct and visualize bibliometric maps while extracting key information from extensive publications. It was utilized for analyzing co-occurrence networks among countries, institutions, and authors, as well as conducting keyword co-occurrence network analysis and density analysis. CiteSpace is another application supporting visual exploration of knowledge maps within literature databases. It provides keyword and citation burst analysis over specific time periods, aiding in the identification of research trends and hotspots within the field. Additionally, Scimago Graphica was employed to visualize the frequency of inter-country collaborations. Finally, Microsoft Office Excel 2021 software was utilized for quantitative analysis of all literature.

## Results

### Quantitative analysis of publication

**FIGURE 2:**
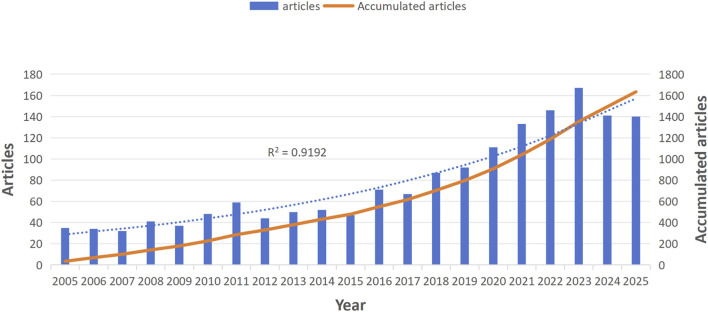
Number of publications per year and the cumulative number.

Based on the search terms, 1,634 articles were identified in WoSCC, including 1,295 articles and 339 review articles. Figure 2 displays the annual and cumulative publication counts related to the topic. From 35 articles in 2005 to 140 in 2025, the annual output showed steady growth in the early years. However, starting in 2020, a significant increase occurred, with publication volumes exceeding 100 articles, indicating heightened interest in this subject. The year 2023 saw the highest output with 167 articles published.

### Analysis of national publication

**FIGURE 3:**
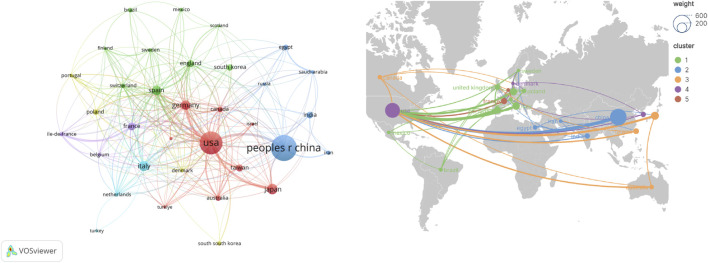
Network of cooperation in each country.

These publications originate from 92 countries or regions. Table 1 lists the top 10 countries by publication volume, with six of them located in Europe and the Americas. Publications from the top three countries account for over sixty percent of the total (n = 1020, 62.4%), including China in Asia (n = 500, 30.6%), the United States in North America (n = 405, 24.8%), and Japan in Asia (n = 115, 7.0%). Using VOSviewer to filter and visualize countries with 10 or more publications, Figure 3 presents each country’s collaborative network based on publication volume and relationships.

**TABLE 1:** Top 10 countries for publications.

Rank	Country	Article	Citations	Total link strength
1	Peoples Rpublic of China	500	16211	101
2	United States	405	29966	326
3	Japan	115	4756	75
4	Italy	107	5646	112
5	Germany	95	5778	132
6	Spain	92	5408	132
7	France	67	5509	101
8	Taiwan	60	1643	36
9	England	59	5038	84
10	India	51	1675	20

### Analysis of institution publications

**FIGURE 4:**
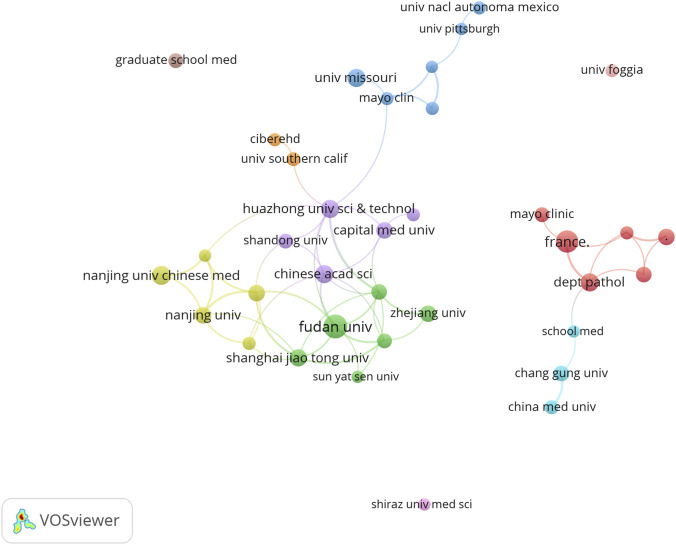
Key institutions and their relationships in this area.

The relevant research was conducted across approximately 2858 institutions worldwide. Table 2 lists the top 10 institutions by publication output. Spain’s Centro de Investigación Biomédica en Red (CIBER) published the highest number of papers at 31, followed by the University of California System from the United States and France’s Institut National de la Santé et de la Recherche Médicale (Inserm), both with 29 publications. Two of the top five institutions are based in China. Figure 4 illustrates the major institutions and their collaborative relationships within this field.

**TABLE 2:** Top 10 institutions for literature output.

Rank	Organization	Country	Article	H-index	Citations
1	Centro de Investigacion biomedica en Red (CIBER)	Spain	31	20	1791
2	University of California system	United States	29	23	2674
3	Institut national de la Sante et de la Recherche Medicale (Inserm)	France	29	25	2931
4	Fudan University	Peoples Rpublic of China	27	13	969
5	Chinese Academy of sciences	Peoples Rpublic of China	25	11	794
6	Mayo clinic	United States	24	10	1253
7	University of Barcelona	Spain	23	17	1845
8	CIBEREHD	Spain	23	15	1168
9	US Department of Veterans Affairs	United States	22	17	1547
10	Chang Gung Memorial hospital	Taiwan	21	11	550

### Analysis of journals

A total of 753 journals have published articles on research in the field of hepatic fibrosis and mitochondrial mechanisms. Table 3 lists the top 10 journals ranked by publication volume, with the International Journal of Molecular Sciences leading with 36 articles. Hepatology ranks second with 28 articles and is also the most highly cited journal.

**TABLE 3:** The top 10 journals were ranked by total citations.

Rank	Journal	Article	2024IF	JCR-c	Total citations	H-index
1	INTERNATIONAL JOURNAL OF MOLECULAR SCIENCES	36	4.9	Q1	1513	19
2	HEPATOLOGY	28	16.8	Q1	1991	19
3	WORLD JOURNAL OF GASTROENTEROLOGY	26	5.4	Q1	1197	18
4	CELLS	23	5.2	Q2	839	12
5	SCIENTIFIC REPORTS	17	3.9	Q1	386	10
6	JOURNAL OF HEPATOLOGY	17	33.0	Q1	1706	17
7	FREE RADICAL BIOLOGY AND MEDICINE	16	8.2	Q1	739	8
8	PLOS ONE	15	2.6	Q2	361	5
9	NUTRIENTS	15	5.0	Q1	407	6
10	FRONTIERS IN PHARMACOLOGY	14	4.8	Q1	340	11

### Analysis of author influence and collaboration

**FIGURE 5:**
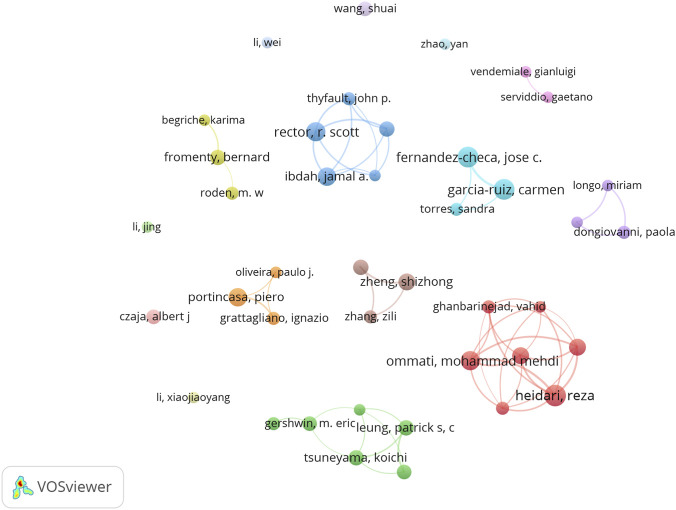
Collaborative relationships among researchers.

A total of 11,166 authors participated in research related to these mechanisms. Table 4 lists the top 10 authors by publication volume. All top 10 authors published at least eight papers. Heidari, Reza ranked first with 13 papers, an h-index of 9, 382 total citations, and a total connection strength of 40. Second is Rector, R Scott, with 12 publications, an h-index of 9, 1,276 total citations, and a total connection strength of 19. We constructed a collaboration network based on 41 authors with at least five publications. Figure 5 illustrates the researchers' collaborative relationships, where node size represents publication volume, color indicates publication time, and line thickness reflects collaboration frequency. This reveals that the primary authors have formed eight major collaborative groups.

**TABLE 4:** The top 10 authors in the number of publications.

Rank	Author	Country	Article	H-index	Citations	Total link strength
1	Heidari, Reza	Iran	13	9	382	40
2	Rector, R Scott	United States	12	9	1276	19
3	Ommati, Mohammad Mehdi	Peoples Rpublic of China	11	8	335	35
4	Garcia-ruiz, Carmen	Spain	10	7	937	16
5	Fernandez-checa, Jose C	Spain	10	9	937	16
6	Ibdah, Jamal A	United States	10	9	1109	15
7	Azarpira, Negar	Iran	9	5	300	31
8	Niknahad, hossein	Iran	9	6	262	29
9	Portincasa, Piero	Italy	9	9	603	6
10	Gores, Gregory J	United States	8	6	936	2

### Co-cited references and bursts detection

Table 5 displays the top 10 most locally cited publications, led by Mansouri A (2018) (Mansouri et al., 2018) and Friedman SL (2018) (Friedman et al., 2018), with 53 and 25 local citations respectively. These studies groundbreakingly demonstrate that in chronic liver disease, mitochondria are not merely victims of energy metabolism but active signaling hubs driving fibrosis progression. This mechanistic understanding provides a core theoretical basis for targeting metabolic regulation, mitochondrial protection, and antifibrotic strategies as therapeutic approaches.

**TABLE 5:** The top 10 most local cited publications.

Rank	First author	Year	Journal	Paper	DOI	Co-citation
1	Mansouri A	2018	GASTROENTEROLOGY	Mitochondrial dysfunction and signaling in chronic liver diseases	10.1053/j.gastro.2018.06.083	53
2	Friedman SL	2018	NAT MED	Mechanisms of NAFLD development and therapeutic strategies	10.1038/s41591-018-0104–9	25
3	Chen Z	2020	FREE RADICAL BIO MED	Role of oxidative stress in the pathogenesis of nonalcoholic fatty liver disease	10.1016/j.freeradbiomed.2020.02.025	24
4	Kisseleva T	2021	NAT REV GASTRO HEPAT	Molecular and cellular mechanisms of liver fibrosis and its regression	10.1038/s41575-020-00372–7	22
5	Eslam M	2020	J HEPATOL	A new definition for metabolic dysfunction-associated fatty liver disease: An international expert consensus statement	10.1016/j.jhep.2020.03.039	22
6	Simoes ICM	2018	INT J BIOCHEM CELL B	Mitochondria in non-alcoholic fatty liver disease	10.1016/j.biocel.2017.12.019	20
7	Chalasani N	2018	HEPATOLOGY	The diagnosis and management of nonalcoholic fatty liver disease: Practice guidance from the American association for the study of liver diseases	10.1002/hep.29367	20
8	Moore MP	2022	HEPATOLOGY	Compromised hepatic mitochondrial fatty acid oxidation and reduced markers of mitochondrial turnover in human NAFLD	10.1002/hep.32324	20
9	Younossi Z	2018	NAT REV GASTRO HEPAT	Global burden of NAFLD and NASH: Trends, predictions, risk factors and prevention	10.1038/nrgastro.2017.109	19
10	Tsuchida T	2017	NAT REV GASTRO HEPAT	Mechanisms of hepatic stellate cell activation	10.1038/nrgastro.2017.38	19

**FIGURE 6:**
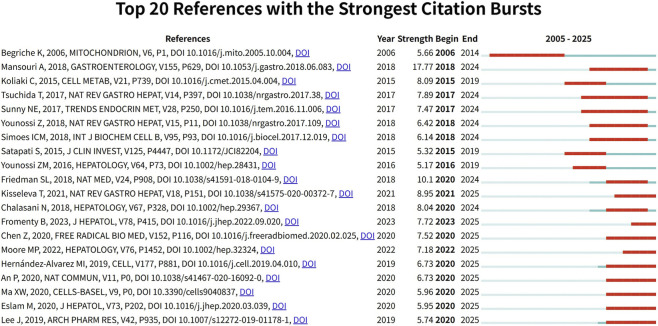
The top 20 references with the strongest citation outbreak.

A citation explosion denotes a surge in citation frequency following a paper’s publication, indicating heightened research interest in the subject. Figure 6 displays the top 20 references with the strongest citation explosions, where the dark blue line represents citation frequency from 2005 to 2025, and the red line indicates the range of the citation surge. The article “Mitochondrial Dysfunction and Signaling in Chronic Liver Diseases” (Mansouri et al., 2018), published in Gastroenterology, exhibited the highest citation burst value from 2005 to 2025, with strength = 17.77 and burst period = 2018–2024. The second strongest citation burst was for the paper titled “Mechanisms of NAFLD development and therapeutic strategies” (Friedman et al., 2018), published in Nature Medicine (strength = 10.1, burst period = 2020–2024). Among the top 20 papers, nine are still experiencing citation bursts, with research primarily focused on mechanisms where metabolic dysfunction drives the progression of fatty liver disease to hepatic fibrosis.

### keyword analysis and analysis of keywords bursts

**FIGURE 7:**
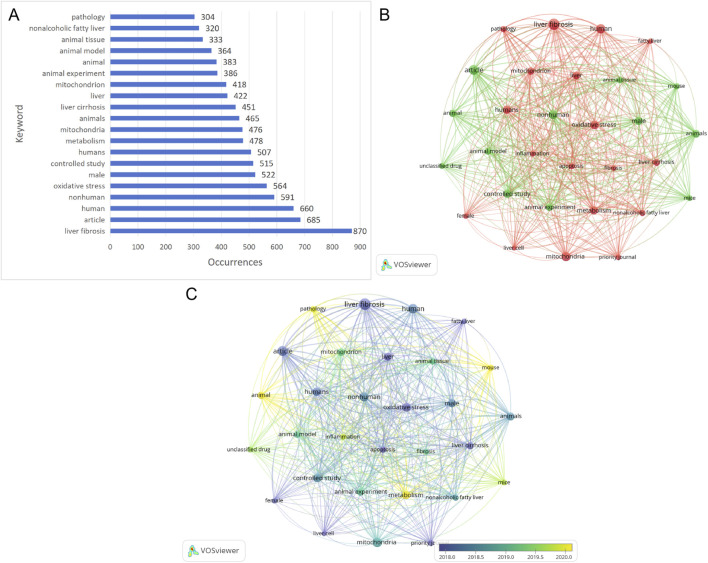
**(A)** A list of the 20 most frequently used keywords. **(B)** Keyword co-occurrence network. **(C)** Time-overlapping co-occurrence analysis network of keywords.

**FIGURE 8:**
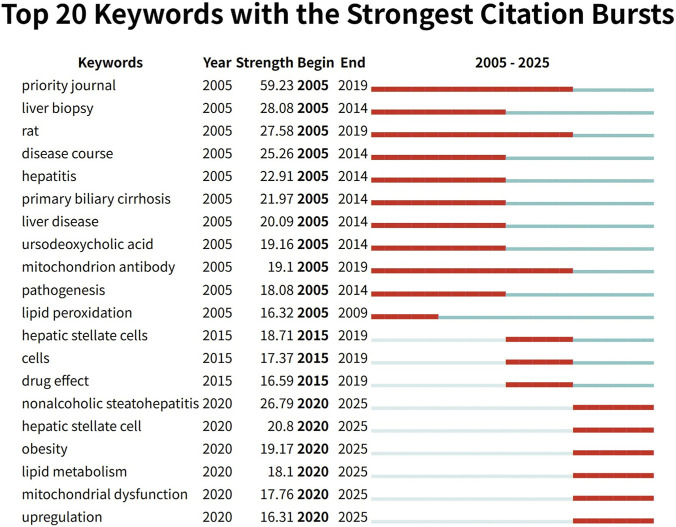
The top 20 keywords with the strongest citation outbreak.

Keyword co-occurrence analysis helps identify relevant research hotspots. Figure 7A displays the top 20 keywords sorted by frequency. The most frequently occurring keyword is “hepatic fibrosis,” appearing 870 times. Among the 1,634 papers, 30 keywords with frequencies greater than or equal to 250 were extracted and clustered. Figure 7B presents a network visualization of these keywords. Node size reflects keyword frequency, with terms grouped into two clusters. A subsequent interpretative synthesis of these clusters, combined with insights from the analysis of highly-cited literature and citation bursts, allowed us to distill three broader, interconnected research themes that characterize the field’s current hotspots: the activation of hepatic stellate cells, the imbalance in mitochondrial quality control, and the vicious cycle of oxidative stress. Figure 7C visualizes temporal overlap among keywords. From cool colors to warm colors, it represents that the average year when the keywords appear increases, and the size of the nodes still represents the frequency, thereby visually presenting the historical evolution of the research hotspots. Figure 8 highlights the top 20 keywords with the strongest bursts. The keyword “priority journal” received the most attention, with strength = 59.23 and burst period = 2005–2019. Next is “liver biopsy,” with strength = 28.08 and burst period = 2005–2014. Recently, keywords such as “nonalcoholic steatohepatitis” (2020–2025), “hepatic stellate cells” (2020–2025), “lipid metabolism” (2020–2025), and “mitochondrial dysfunction” (2020–2025) are currently experiencing burst periods of usage, indicating that future research focus will remain concentrated on these keywords.

The keyword “priority journal,” which exhibited the strongest citation burst, is a non-substantive indexing tag occasionally assigned by literature databases and does not reflect a thematic research focus. Its prominence in the burst analysis is an artifact of database indexing practices prevalent in earlier periods of the literature covered by this study. Manual verification confirmed that publications associated with this tag were not systematically relevant to the core topic of mitochondrial mechanisms in hepatic fibrosis. This finding underscores the importance of contextual interpretation of algorithmic bibliometric outputs. The thematic keywords discussed above were analyzed after excluding such non-substantive indexing interference to ensure the robustness of the hotspot identification.

## Discussion

### Principal findings

This study employed bibliometric methods to systematically analyze research trends on mitochondrial mechanisms in hepatic fibrosis from 2005 to 2025. Results indicate sustained rapid growth in research output, with annual publications increasing from 35 in 2005 to 140 in 2025. Publication rates accelerated significantly after 2020, consistently exceeding 100 papers annually. This trend may be driven by multiple factors: First, the global prevalence of metabolic liver diseases, exemplified by NASH, has surged dramatically (Hye Khan et al., 2019). Its core pathophysiological mechanism—a vicious cycle of lipid metabolism disorders and mitochondrial dysfunction—has become an urgent scientific and clinical challenge. Second, technological advances—such as high-resolution mitochondrial function assays, metabolomics, and single-cell sequencing—have enabled deeper cellular and molecular insights into mitochondrial roles in fibrosis. Finally, a paradigm shift in hepatology research, moving from late-stage cirrhosis to reversible interventions in early fibrosis, has spurred exploration of mitochondrial-targeted prevention and therapeutic strategies (Zhang et al., 2017).

Analysis of 1,634 global publications reveals highly internationalized hepatic fibrosis research, with China and the United States forming the core of collaborative networks. Current consensus emphasizes mitochondria as a key signaling source driving hepatic fibrosis. Current research frontiers focus on metabolic liver diseases, such as NASH, focusing on lipid overload-induced mitochondrial dysfunction that propels fibrosis through mechanisms like hepatic stellate cells activation.

Analysis of countries and regions confirms the uneven distribution of global research capabilities and the inevitability of collaboration. The United States leads in output and influence due to its long-term investment and robust basic research infrastructure. China’s rapid growth in recent years reflects increased resource allocation and accelerated advancement in liver disease research. However, international collaboration networks indicate that partnerships between the US and Europe, as well as between the US and China, are pivotal for driving major breakthroughs. This suggests that regions with relatively limited resources should actively integrate into international collaborations to enhance their research competitiveness.

Analysis of institutional collaboration networks reveals a clustered cooperation model centered around a few top-tier research institutions. These core institutions serve not only as hubs for knowledge production but also as nexuses for the convergence of talent, technology, and ideas. For instance, CIBER’s pivotal role in liver disease research across Spain and Europe exemplifies the advantages of national research consortia in pooling resources and focusing on major research challenges.

Network analysis of author collaborations reveals that multiple active and closely connected academic communities have emerged within this field. Highly productive authors often serve as core nodes within these communities, with their work driving the advancement of specific research directions such as mitophagy and redox biology. Notably, Asian authors are relatively underrepresented among the top high-output researchers, contrasting with the high overall publication volume from Asian countries (particularly China). This suggests Asian research may still have room for improvement in pioneering original core theories and cultivating international academic leaders.

Regarding journal impact, Q1-ranked journals dominate the top ten most-cited publications, indicating that high-quality research gravitates toward high-impact platforms. Although China is a major contributor to global research output, no Asian-based journal has yet entered the top ten. This reflects the historical inertia of the current global academic publishing landscape and presents a challenge for China in cultivating domestically hosted flagship journals with international influence.

Analysis of highly cited and explosive publications not only identifies landmark studies but also reveals the evolutionary trajectory of domain knowledge. The most frequently cited works, such as Mansouri A, 2018; Friedman SL, 2018, established the paradigm of mitochondrial dysfunction as a common mechanism and therapeutic target in chronic liver disease. The recent citation surge centers on NASH mechanisms and therapies, indicating the field’s full-scale engagement with this foremost contemporary challenge in hepatology. Collectively, these publications form a knowledge chain progressing from “phenomenon description” to “mechanism elucidation” and ultimately to “translational exploration.”

Our bibliometric data delineate a clear evolutionary trajectory in this field. The early phase, highlighted by keyword bursts such as “liver biopsy” and “disease course” was predominantly descriptive, focusing on clinical and histopathological phenotyping. Subsequently, the research paradigm shifted decisively toward mechanistic dissection, as evidenced by the sustained prominence of keywords like “oxidative stress””apoptosis” and “mitophagy” This period correlated with seminal, highly-cited reviews that established mitochondrial dysfunction as a common pathogenic driver. Currently, the frontier is characterized by a translational and integrative focus. The strong recent bursts of keywords including “nonalcoholic steatohepatitis” “lipid metabolism” and “biomarker” alongside the rising citation impact of studies on therapeutic strategies, signal a maturation of the field. The goal is no longer merely to describe the mechanism but to leverage this understanding for patient stratification, targeted interventions, and the development of mitochondrial-focused therapeutics within the complex landscape of metabolic liver disease. This trajectory—from phenotype to mechanism to translation—frames the future agenda for research on mitochondrial dysfunction in hepatic fibrosis.

### The molecular mechanism of mitochondrial regulation in liver fibrosis

The concept that mitochondrial dysfunction and metabolic reprogramming drive hepatic fibrosis is scientifically grounded in the renewed recognition of mitochondria’s central role in cellular stress responses. Under physiological conditions, mitochondria efficiently generate ATP through oxidative phosphorylation while finely regulating reactive oxygen species levels, calcium homeostasis, and apoptotic signaling (Niemi et al., 2019). However, in the context of chronic liver injury, particularly characterized by lipotoxicity, mitochondria suffer dual functional and structural impairments (Chen et al., 2018; Tao et al., 2021).

The pivotal mechanism involves: excessive free fatty acids flooding into mitochondria for β-oxidation, overloading the electron transport chain and triggering massive ROS production, thereby inducing oxidative stress. Concurrently, damaged mitochondria release molecules such as mitochondrial DNA and cytochrome C, directly activating inflammasomes or inducing apoptosis. These events collectively constitute a damaging microenvironment. Within this environment, hepatic stellate cells—the core effector cells of hepatic fibrosis—become activated (Chen et al., 2019; Yang et al., 2017). Activated hepatic stellate cells themselves undergo profound mitochondrial metabolic reprogramming, shifting their energy source from fatty acid oxidation to aerobic glycolysis to meet the bioenergetic and biosynthetic demands of their proliferation and the synthesis of large amounts of extracellular matrix.

Furthermore, the failure of mitophagy leads to the accumulation of dysfunctional mitochondria, further amplifying damage signals and forming a vicious cycle of “dysfunction-insufficient clearance-more damage” (Chatterjee et al., 2022). Recent research has significantly deepened this mechanistic understanding, revealing a sophisticated, multi-layered regulatory network that extends beyond oxidative stress and metabolic imbalance. This network is now understood to encompass dynamic mitochondrial quality control (MQC), epigenetic reprogramming, and intricate non-coding RNA networks, which collectively dictate the fibrotic fate of the liver.

Mitochondrial homeostasis is maintained through a balance of fission, fusion, mitophagy, and biogenesis (Furu et al., 2024). In chronic liver injury, this balance is disrupted. Excessive mitochondrial fission, mediated by increased Drp1 activity, generates a pool of fragmented, dysfunctional mitochondria that are prone to ROS overproduction and are inefficient at ATP generation (Ma et al., 2023). Conversely, the processes of fusion and mitophagy are often impaired, leading to the accumulation of damaged organelles that perpetuate injury signals and inflammatory responses across hepatocytes, hepatic stellate cells, and macrophages. Simultaneously, the downregulation of key biogenesis regulators like PGC-1α compromises the generation of new, healthy mitochondria, exacerbating the cellular bioenergetic crisis (Abu et al., 2023).

Recent research has uncovered sophisticated regulatory layers beyond oxidative stress. Epigenetically, the chromatin remodeler BAZ2B, which is upregulated in human MASH and correlates with fibrosis severity, represses PPARα and other metabolic genes. This repression inhibits mitochondrial fatty acid β-oxidation, and its genetic ablation *in vivo* has been shown to attenuate disease, thereby defining a key BAZ2B to PPARα to mitochondrial function axis (Tu et al., 2025). Concurrently, non-coding RNAs such as miR-29a fine-tune mitochondrial function by targeting multiple pathways (Matsumoto et al., 2016). These actions include improving mitochondrial respiration through suppression of MCJ, a negative regulator of the electron transport chain; inhibiting pro-fibrotic YAP/TAZ signaling; and dampening NLRP3 inflammasome-mediated pyroptosis.

This evolving multi-level mechanistic understanding spans epigenetic, post-transcriptional, and metabolic controls, marking a definitive shift in the field from describing correlations to dissecting causation. It provides a solid foundation for developing next-generation therapies that target these specific nodes, such as BAZ2B inhibitors and miR-29a mimics. Ultimately, this knowledge underscores the critical need for developing combinatorial therapeutic strategies and discover actionable biomarkers to enable personalized intervention in hepatic fibrosis.

### Comparisons with previous studies

Compared with previous bibliometric analyses of hepatic fibrosis mechanisms, this study not only confirms the sustained growth trend in this field and the research landscape centered on the United States and China but also reveals new evolutionary dynamics through multidimensional analysis.This focused scope allows for a more granular examination of the subfield’s evolution. First, by employing a combined WoSCC and Scopus search strategy and a multi-software analytical approach (VOSviewer, CiteSpace), we captured a comprehensive dataset and performed simultaneous analyses of collaboration networks, citation bursts, and keyword evolution that go beyond the descriptive output counts common in earlier studies. Second, our analysis, covering the period up to 2025, precisely captures the recent and decisive shift in research paradigms from fundamental mechanism exploration toward clinical translation, a trend marked by the strong citation bursts of studies on therapeutic strategies and the emergence of keywords like “biomarker” and “nonalcoholic steatohepatitis” as current frontiers. This temporal extension and thematic precision provide a clearer and more updated trajectory than previously available. These findings not only deepen our understanding of the structural evolution within this specific research nexus but also establish clear, data-driven key nodes and priority directions for future academic exploration and clinical translation.

### Strengths and limitations

This study marks the first integrated application of multiple bibliometric software tools—including VOSviewer, CiteSpace, and Scimago Graphica—to conduct a systematic analysis of the mitochondrial and hepatic fibrosis field. It provides clear, visual trend and hotspot maps to guide future research directions in this domain. Second, through detailed analysis of collaboration networks, core journals and high-impact publications, this study enables researchers to rapidly identify key investigators, core academic circles, and essential literature, thereby efficiently grasping the cutting edge of the field. Third, the analysis clearly demonstrates that current research is evolving from fundamental mechanisms (such as oxidative stress, mitophagy) toward clinical translation (such as biomarkers, targeted therapeutic strategies), highlighting a multidisciplinary trend combining metabolic interventions with anti-fibrotic therapies. Finally, this review systematically maps the evidence chain establishing mitochondrial dysfunction as a central hub in hepatic fibrosis. It provides a mechanistic framework for comprehensively understanding this pathological process, potentially aiding future development of targeted, precision therapies that improve clinical management and prognosis of hepatic fibrosis.

This review also has several limitations. First, as a bibliometric analysis focusing on the association between mitochondria and the mechanisms of hepatic fibrosis, data collection and processing relied heavily on software tools such as VOSviewer and CiteSpace. Their algorithms and parameter settings may influence the presentation of knowledge maps. While such analyses cannot fully replace in-depth systematic reviews, they provide an effective approach for grasping macro trends and structures within this field. Second, the data sources for this study were limited to the WoSCC and Scopus databases—a common boundary in bibliometric research. This implies that relevant studies indexed in other databases (such as PubMed, EMBASE) may have been excluded, introducing a risk of omission. Third, the substantial growth in publications from 2005 to 2025 may partly stem from the overall increase in scientific output within liver disease research during this period, rather than solely reflecting the independent developmental intensity of this specific subfield. Finally, due to the delayed accumulation of academic influence, recently published high-quality research may not yet have garnered sufficient citation attention, potentially underrepresenting its significance in this analysis. Future research should continue monitoring this field and promptly update data to incorporate the latest advancements and changes as their impact becomes apparent.

### Clinical implications for clinicians and policymakers

Based on the trends in bibliometrics, a specific transformation roadmap is gradually becoming clear. The recent strong emergence of the keywords “non-alcoholic steatohepatitis” and “lipid metabolism” indicates that metabolic liver diseases are the primary clinical context for applying these research results. In this context, the highly cited mechanism studies have pointed out specific and prioritizable targets. For the development of biomarkers, molecules that directly reflect mitochondrial damage and metabolic status, such as circulating extracellular mitochondrial DNA (mtDNA) and mitochondrial-specific metabolites, are the preferred candidates for technical and clinical validation. For patient stratification, these biomarkers can help define clinically feasible mitochondrial dysfunction phenotypes. Subsequently, treatment efforts should prioritize these stratified patients and use these drugs in trials focusing on the pathways emphasized by the research frontiers, including inducing mitochondrial autophagy to clear damaged organelles, precise antioxidants to alleviate oxidative stress, and metabolic regulators to restore fatty acid oxidation. This series of processes, from biomarker-based stratification classification to mechanism-based intervention measures, directly reflects the evolution of this field from mechanistic discoveries to therapeutic goals, providing a practical path for improving the management of liver fibrosis.

For clinicians, this trend underscores the importance of establishing multidisciplinary teams centered around the “liver-metabolism axis.” Hepatologists, gastroenterologists, pathologists, radiologists, and even clinical pharmacologists must collaborate. This collaboration will enable more precise assessment of mitochondrial dysfunction phenotypes in patients—such as oxidative stress-type or metabolic imbalance-type—transcending conventional etiological treatments to inform personalized combination therapy strategies. Against the backdrop of rapidly advancing therapeutic approaches, clinicians must continuously update their knowledge to master emerging biomarkers for mitochondrial function assessment and potential therapeutic targets, which is crucial for improving patient outcomes.

For policymakers and healthcare system administrators, the insights from this study provide a basis for adjusting medical resource allocation and research funding priorities. Policy frameworks should encourage and fund the translation of mitochondrial function biomarkers into accessible clinical diagnostic tools, integrating them into systems for hepatic fibrosis risk stratification and treatment efficacy monitoring. Resource allocation should prioritize translational research and clinical trials targeting mitochondrial metabolism, autophagy, and redox balance, while fostering interdisciplinary platforms bridging hepatology and metabolic medicine. Additionally, health policies should promote comparative effectiveness research based on mitochondrial phenotypes to accumulate real-world evidence, thereby refining and updating clinical guidelines for hepatic fibrosis management.

In summary, this study offers stakeholders a trajectory of progress and a perspective on future directions within this field. Adopting and integrating these cutting-edge insights holds promise not only for reducing the disease burden of hepatic fibrosis but also for advancing the management model toward greater precision, mechanism-based approaches, and enhanced multidisciplinary collaboration. This compels us to re-evaluate existing clinical pathways and motivates researchers, clinicians, and policymakers to collectively commit to translating the latest evidence from mitochondrial science into tangible clinical benefits, ultimately improving the overall quality of care for patients with hepatic fibrosis.

## Conclusion

In summary, research on the mechanisms underlying mitochondrial involvement in hepatic fibrosis has increasingly become a central topic in hepatology. The significant growth in literature over the past 2 decades reflects the rising importance of this research direction. This study employs bibliometrics and knowledge graph visualization techniques to systematically reveal the developmental trajectory, knowledge structure, and collaborative networks within this field over the past 20 years. Analysis indicates that future research will focus on achieving effective reversal and precise intervention for hepatic fibrosis. The core strategy lies in targeted therapies based on mitochondrial quality control, metabolic reprogramming regulation, and redox balance restoration. This study provides researchers with a clearer understanding of the field’s evolutionary logic and key milestones, laying the groundwork for subsequent in-depth mechanistic exploration and clinical translation research. Future research outcomes are anticipated to empower more effective clinical management of hepatic fibrosis patients, ultimately offering novel breakthrough pathways to halt liver disease progression and improve long-term patient outcomes.

## Data Availability

The datasets presented in this study can be found in online repositories. The names of the repository/repositories and accession number(s) can be found in the article/Supplementary Material.
