# Supplementary material for: Research trends of mitochondrial dysfunction in hepatic fibrosis: a bibliometric analysis
Source: Front Physiol. 2026 Feb 27;17:1767822. doi: 10.3389/fphys.2026.1767822 (PMC12982054; doi:10.3389/fphys.2026.1767822)
Supplement: Supplementary file 1 [file DataSheet1.docx]

**Table S1** Search strategy via Web of Science Core Collection

| **Step** | **Search strategy** |
| --- | --- |
| **#1** | TS=(“Mitochondrion”OR“Mitochondrial Contraction”OR“Contraction, Mitochondrial”OR“Contractions, Mitochondrial”OR“Mitochondrial Contractions”) |
| **#2** | TS=(“Hepatic Fibrosis”OR“Cirrhosis, Liver”OR“Hepatic Cirrhosis”OR“Cirrhosis, Hepatic”OR“Fibrosis, Liver”OR“Liver Fibrosis”) |
| **#3** | #1 AND #2 |

**Table S1** Search strategy via Scopus

| **Step** | **Search strategy** |
| --- | --- |
|  | TITLE-ABS-KEY ( “Mitochondrion”OR“Mitochondrial Contraction”OR“Contraction, Mitochondrial”OR“Contractions, Mitochondrial”OR“Mitochondrial Contractions”) AND TITLE-ABS-KEY ( “Hepatic Fibrosis”OR“Cirrhosis, Liver”OR“Hepatic Cirrhosis”OR“Cirrhosis, Hepatic”OR“Fibrosis, Liver”OR“Liver Fibrosis”) |
| **#1** |  |
|  |  |
